# Corrigendum: A brain-sparing diphtheria toxin for chemical genetic ablation of peripheral cell lineages

**DOI:** 10.1038/ncomms15673

**Published:** 2017-05-17

**Authors:** Mafalda M. A. Pereira, Inês Mahú, Elsa Seixas, Noelia Martinéz-Sánchez, Nadiya Kubasova, Roksana M. Pirzgalska, Paul Cohen, Marcelo O. Dietrich, Miguel López, Gonçalo J. L. Bernardes, Ana I. Domingos

Nature Communications 8: Article number: 14967; DOI: 10.1038/ncomms14967 (2017); Published 04 03 2012; Updated 05 17 2017

The financial support for this Article was not fully acknowledged. The Acknowledgements should have included the following:

[***Human Frontiers Science Program (HFSP) funds the labs of A.I.D. and P.C. ***].

