# Supplementary material for: A brain-sparing diphtheria toxin for chemical genetic ablation of peripheral cell lineages
Source: Nat Commun. 2017 Apr 3;8:14967. doi: 10.1038/ncomms14967 (PMC5382263; doi:10.1038/ncomms14967)
Supplement: Supplementary Information — Supplementary Figures, Supplementary Table. [file ncomms14967-s1.pdf]

Supplementary Figure 1

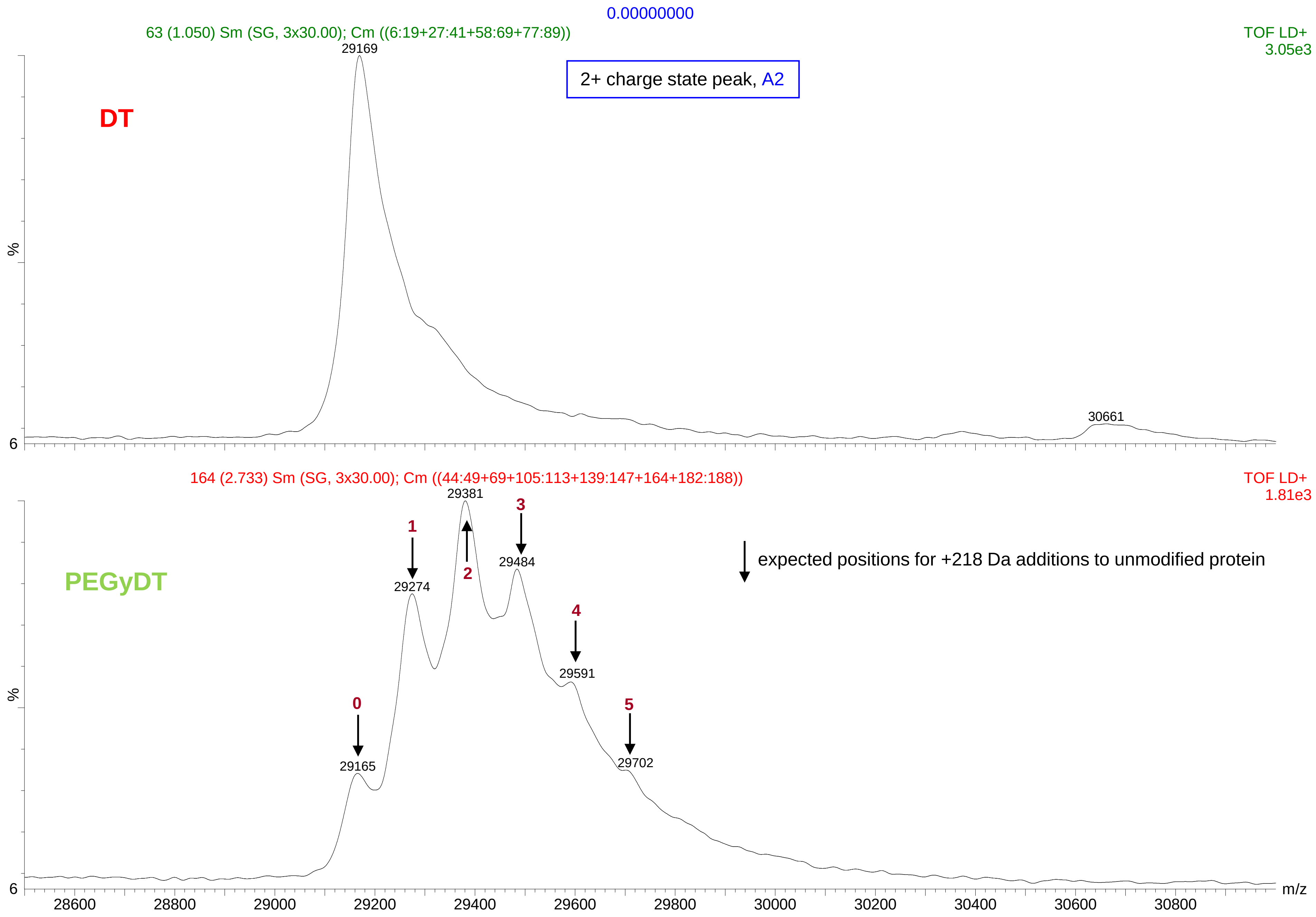

Supplementary Figure 1 – Mass spectrometry raw data (related to Figure 1).

Supplementary Figure 2

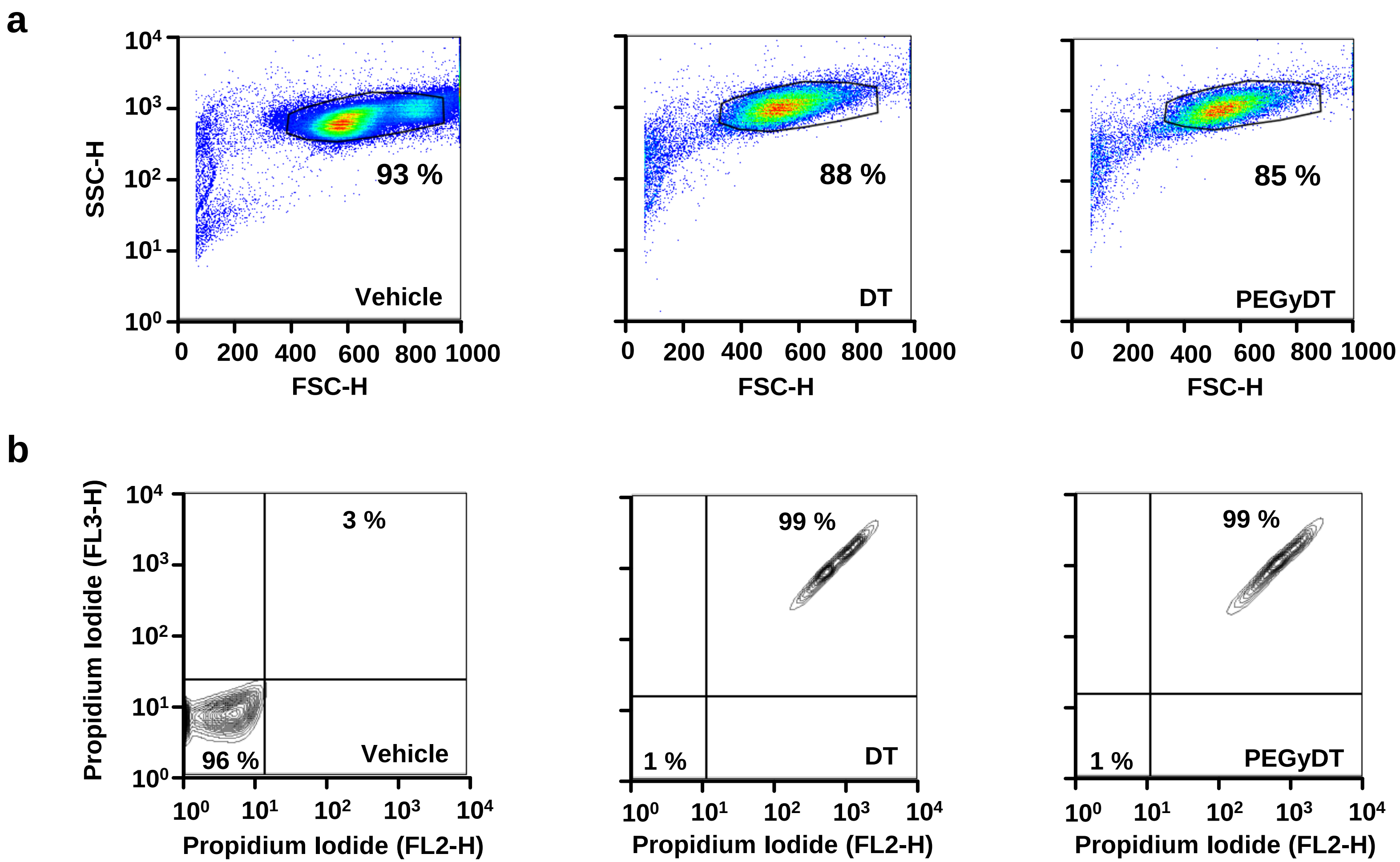

Supplementary Figure 2 – DT retains *in vitro* functionality after modification. a) Gating strategy of FACS data obtained using HeLa cells for vehicle; DT and PEGyDT after 48 h of incubation (representative of 3 experiments); **b)** Representative of 3 experiments contour plots of vehicle, DT and PEGyDT populations after 48 h of incubation on HeLa cells (related to Figure 1).

Supplementary Figure 3

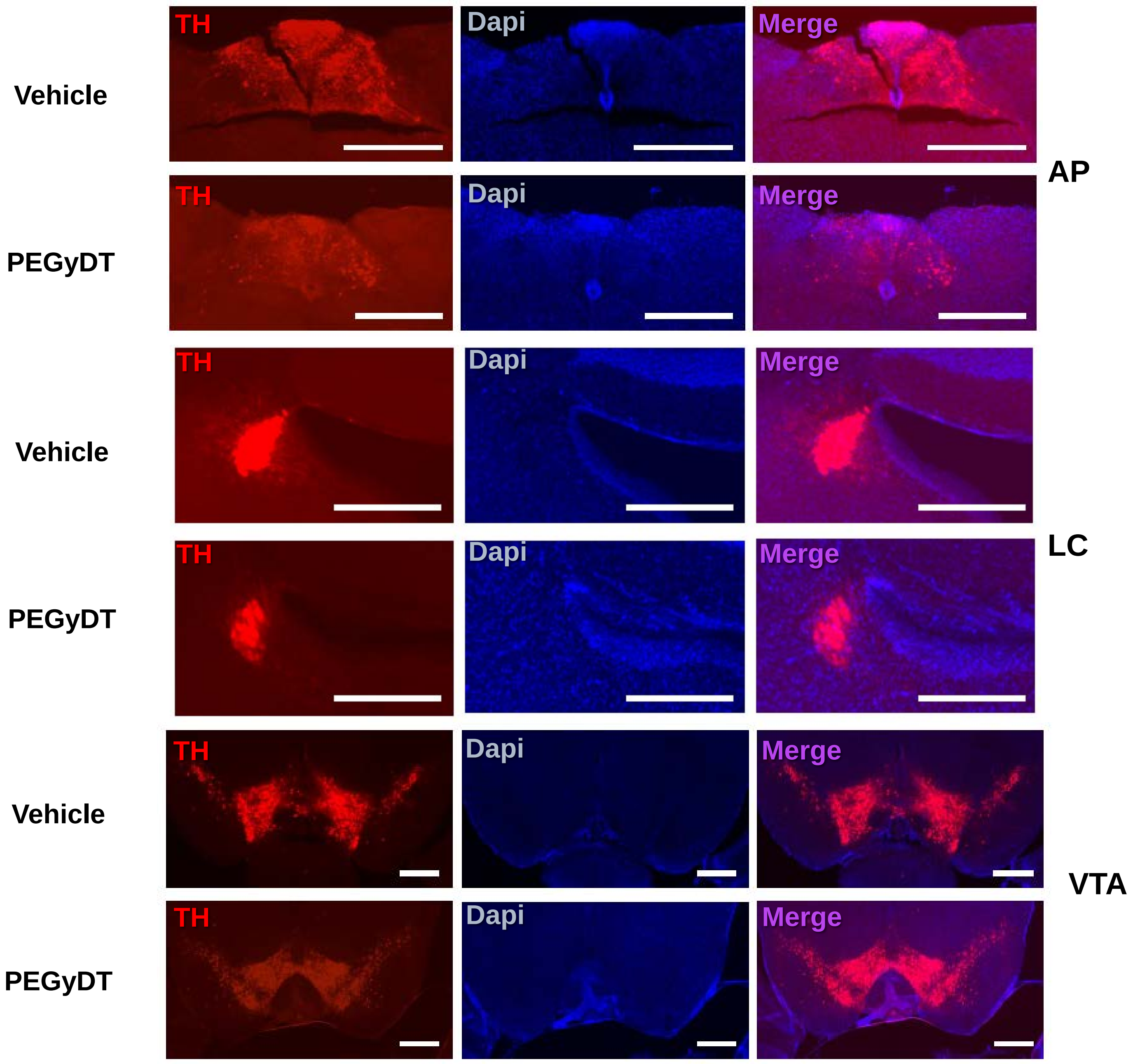

Supplementary Figure 3 (cont)

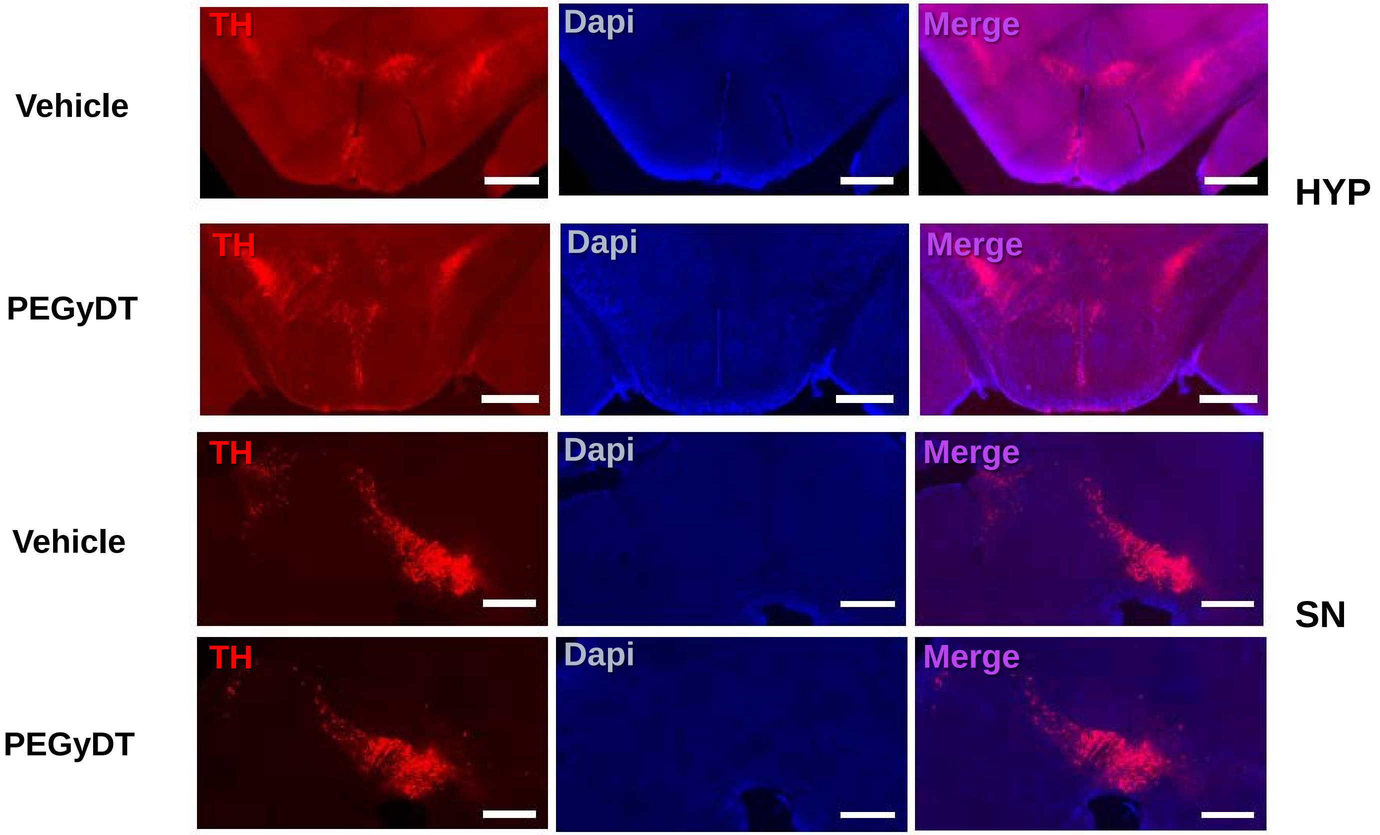

Supplementary Figure 3 – BRAINSPAReDT does not ablate neurons in the brain. Confocal microscopy imaging of TH^{+} neurons after ip injection of PEGyDT or vehicle. Scale bar is 500 µm for area postrema (AP), locus coeruleus (LC), ventral tegmental area (VTA), hypothalamus (HYP) and saggital plane of substantia nigra (SN) (related to Figure 2).

Supplementary Figure 4

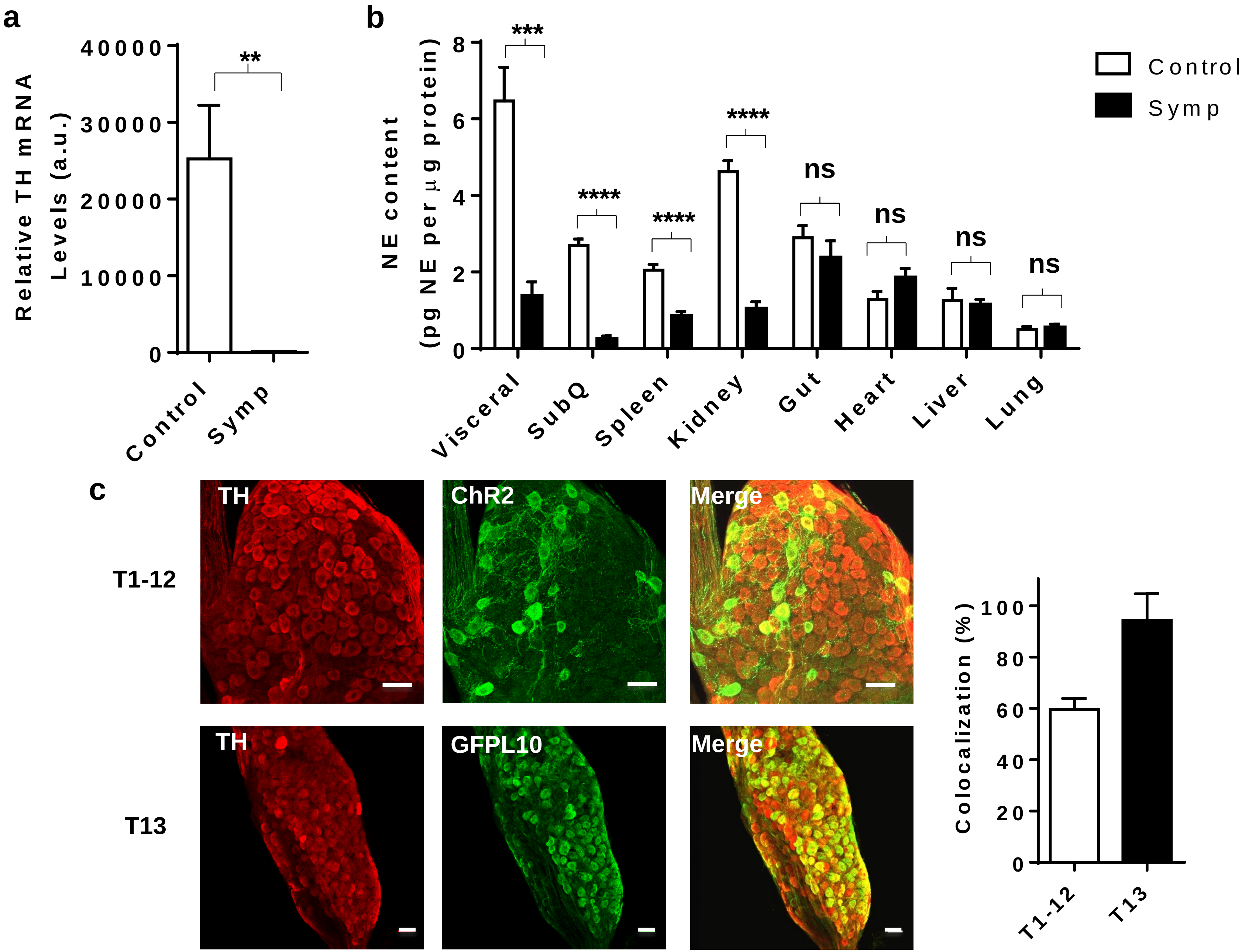

Supplementary Figure 4 – BRAINSPAReDT leads to decreased TH mRNA levels in sympathetic ganglia and decreased NE levels in kidney and spleen. a) TH mRNA levels in superior cervical ganglia (** $p < 0.001$, $n = 9$); **b)** NE content in visceral adipose tissue, SubQ adipose tissue, spleen, kidney, gut, heart, liver and lung (*** $p < 0.0001$, **** $p < 0.00001$, $n = 6$); **c)** Confocal microscopy imaging of the thoracic ganglia (T1-T12 and T13) of the sympathetic chain and respective colocalization. Ganglia T1-12 were dissected from TH-Cre; LSL-ChR2-YFP mice and T13 was dissected from TH-Cre; LSL-GFPL10 mice. Scale bar is 50 µm. Statistics were performed using unpaired t-test. Data are represented as mean ± SEM (related to Figure 2 and Figure 4).

Supplementary Figure 5

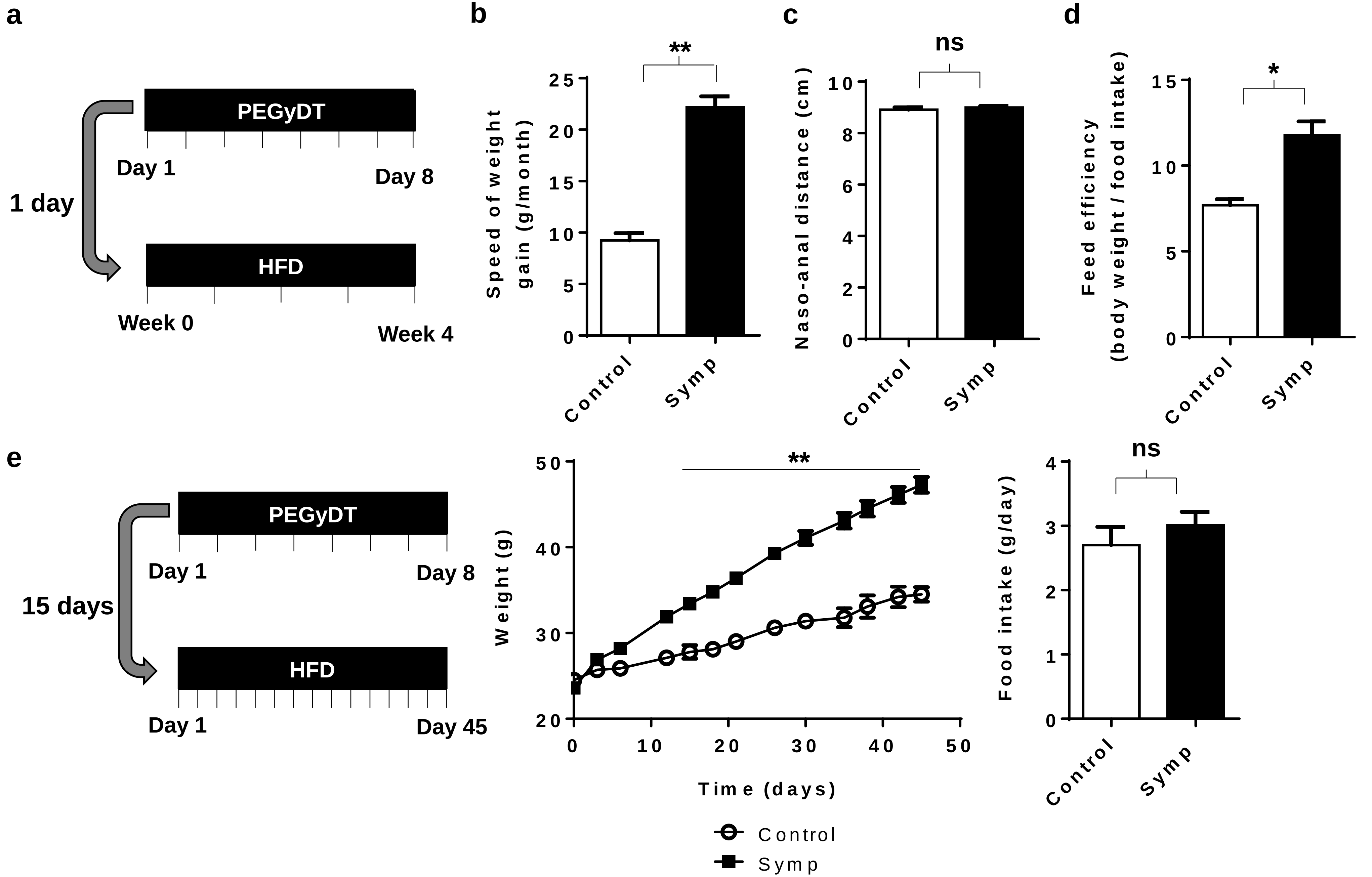

Supplementary Figure 5 – Sympathectomy with BRAINSPAReDT predisposes mice to obesity independently of food intake. a) Schematic timeline representation of PEGyDT injections and HFD period; **b)** Speed of weight gain during HFD regimen (** $p < 0.001$, n = 6); **c)** Naso-anal distance during HFD regimen (n = 6); **d)** Feed efficiency (* $p < 0.01$, n = 4); **e)** Schematic timeline representation of PEGyDT injections and HFD period, weight gain and food intake when HFD regimen starts 2 weeks after regional SNS ablation (** $p < 0.001$, n = 6). Statistics were performed using unpaired t-test. Data are represented as mean ± SEM (related to Figure 5).

Supplementary Figure 6

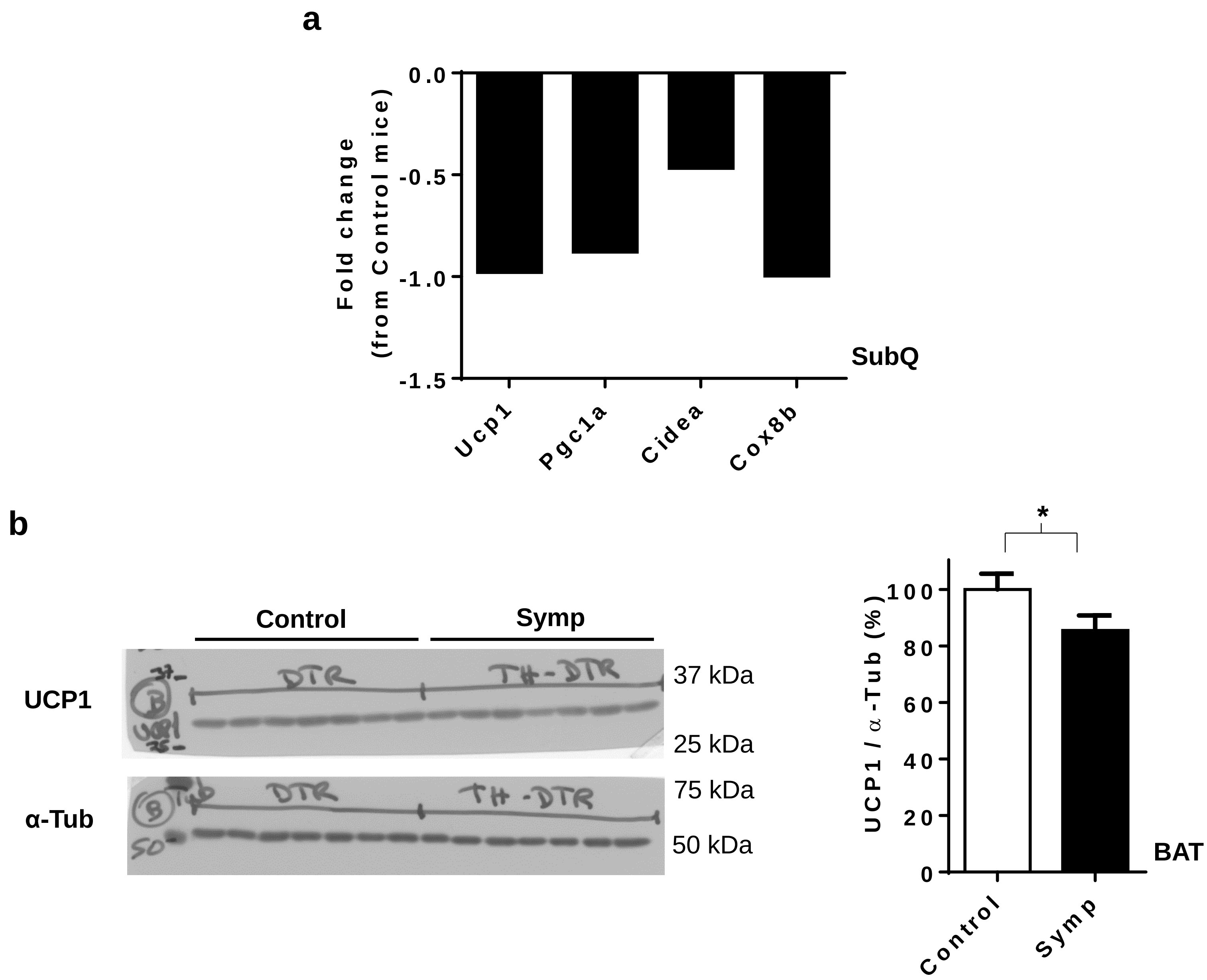

Supplementary Figure 6 – Obese mice sympathectomized with BRAINSPAReDT have low NE content in SubQ adipose tissue and a deficient thermogenic molecular profile. a) Fold change of mRNA levels relative to control mice (n = 6); **b)** UCP1 levels in BAT representative of 3 blots (* $p < 0.01$, n = 7 for Symp and n = 8 for Control). Statistics were performed using unpaired t-test. Data are represented as mean ± SEM (related to Figure 6).

Supplementary Figure 7

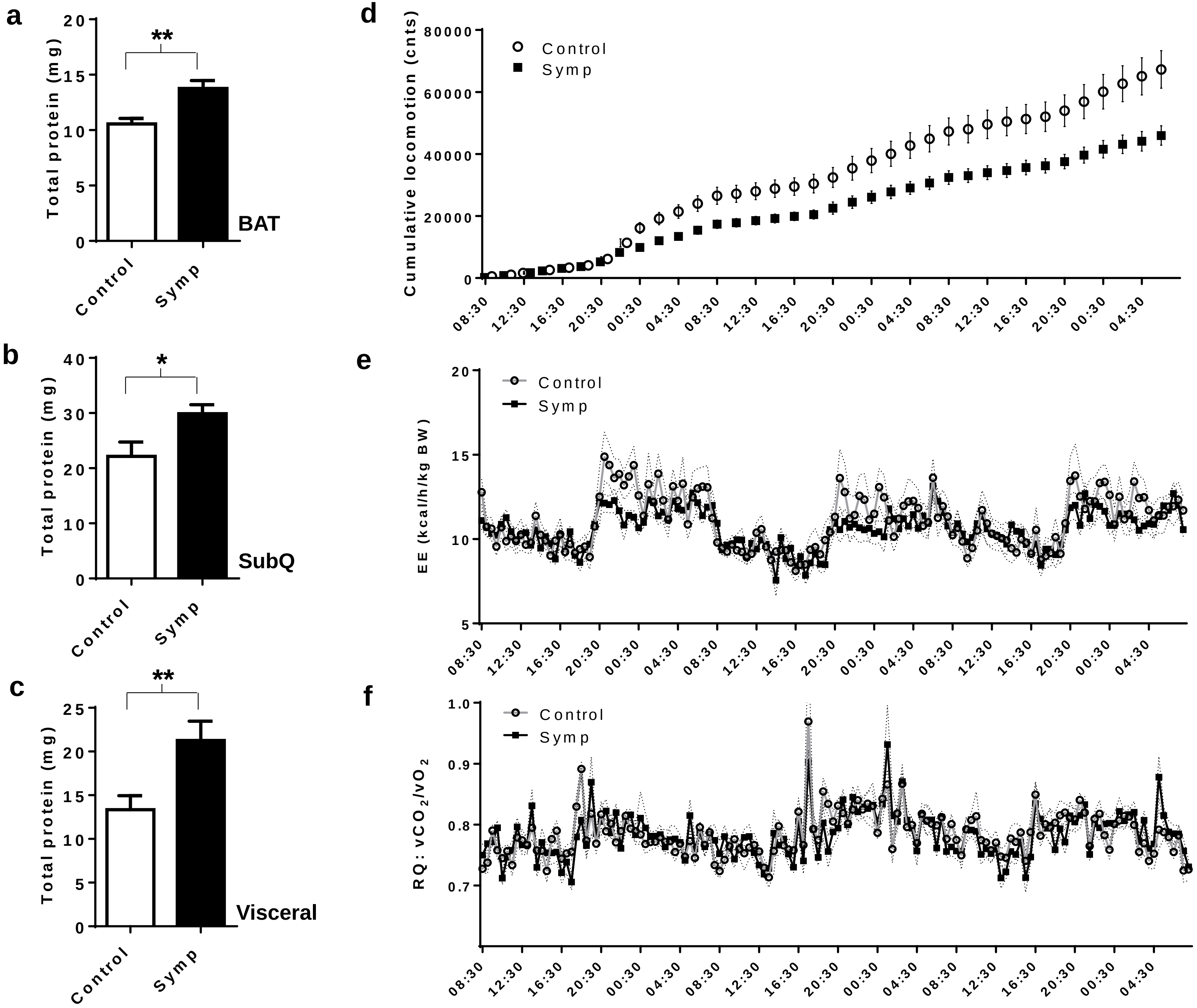

Supplementary Figure 7 – Mice sympathectomized with BRAINSPAReDT present hypoactivity but normal energy expenditure and respiratory quotient. a) Protein quantification in BAT; **b)** Protein quantification in SubQ; **c**) Protein quantification in visceral WAT; **d)** Cumulative locomotion (n = 8); **e)** Energy expenditure (n = 8); **f)** Respiratory quotient (n = 8). Statistics were performed using unpaired t-test. Data are represented as mean ± SEM (related to Figure 6).

Supplementary Table 1

Primers used for quantitative PCR			
Tissue	Gene	Forward Primer	Reverse Primer
SubQ and SCG	TH	GGTATACGCC ACGCTGAAGG	TAGCCACAGTA CCGTTCCAGA
	Ucp1	ACTGCCACAC CTCCAGTCATT	CTTTGCCTCAC TCAGGATTGG
	Pgc1a	CCCTGCCATT GTTAAGACC	TGCTGCTGTTC CTGTTTTC
	Cidea	TGCTCTTCTGT ATCGCCCAGT	GCCGTGTTAAG GAATCTGCTG
	Cox8b	GAACCATGAAG CCAACGACT	GCGAAGTTCAC AGTGGTTCC
	GAPDH	AACTTTGGCAT TGTGGAAGG	ACACATTGGGG GTAGGAACA
BAT	Ucp1	CGATGTCCATGT ACACCAAGGAA	GACCCGAGTCG CAGAAAAGAA
	Pgc1a	CGATCACCATAT TCCAGGTCAG	CGATGTGTGCG GTGTCTGTAGT
	Cidea	CCTACGACATCC GATGCACAA	TCTGTGCAGCAT AGGACATAAACC
	HPRT	AGCCGACCGGTT CTGTCAT	GGTCATAACCTG GTTCATCATCAC

Supplementary Table 1 – List of primers used for Quantitative PCR.
